# Genetic evaluation of eggshell color based on additive and dominance models in laying hens

**DOI:** 10.5713/ajas.19.0345

**Published:** 2019-08-26

**Authors:** Jun Guo, Kehua Wang, Liang Qu, Taocun Dou, Meng Ma, Manman Shen, Yuping Hu

**Affiliations:** 1 Jiangsu Institute of Poultry Science, Yangzhou, Jiangsu, 225125, China

**Keywords:** Bayesian, Eggshell Color, Genetic Evaluation, Heritability, Layer

## Abstract

**Objective:**

Eggshells with a uniform color and intensity are important for egg production because many consumers assess the quality of an egg according to the shell color. In the present study, we evaluated the influence of dominant effects on the variations in eggshell color after 32 weeks in a crossbred population.

**Methods:**

This study was conducted using 7,878 eggshell records from 2,626 hens. Heritability was estimated using a univariate animal model, which included inbreeding coefficients as a fixed effect and animal additive genetic, dominant genetic, and residuals as random effects. Genetic correlations were obtained using a bivariate animal model. The optimal diagnostic criteria identified in this study were: L* value (lightness) using a dominance model, and a* (redness), and b* (yellowness) value using an additive model.

**Results:**

The estimated heritabilities were 0.65 for shell lightness, 0.42 for redness, and 0.60 for yellowness. The dominance heritability was 0.23 for lightness. The estimated genetic correlations were 0.61 between lightness and redness, −0.84 between lightness and yellowness, and −0.39 between redness and yellowness.

**Conclusion:**

These results indicate that dominant genetic effects could help to explain the phenotypic variance in eggshell color, especially based on data from blue-shelled chickens. Considering the dominant genetic variation identified for shell color, this variation should be employed to produce blue eggs for commercial purposes using a planned mating system.

## INTRODUCTION

Eggshells with a uniform color and intensity are important for egg production because many consumers assess the quality of an egg according to the shell color. The influence of genetics on avian eggshell color has been known for many years, and there are differences in the causes and functions of egg color variations [1–3]. Estimating the variance components of eggshell color is helpful for assessing the genetic potential of the crossbred population of native blue-shelled chickens. In practical terms, eggshell color is one of the key factors that affect consumer acceptance. Intense and uniform egg colors have been selected in commercial white and brown shelled layers for several decades [4,5]. The shell color has not been selected intensively in blue-shelled chickens. Determining the variability in genetic dominance would increase the accuracy of narrow heritability and selection in mating systems.

The pigments responsible for eggshell color are mainly protoporphyrins and biliverdins. Protoporphyrin IX is the most common pigment and it produces red, brown, and black colorations, whereas biliverdin and its zinc chelate yield blue and blue-green colorations [6–8]. Many regulators are presumably involved in the synthesis, transportation, and release of these pigments. Numerous studies have indicated that dominant genetic factors are involved with eggshell coloration. Hutt [9] suggested that mutations in one of many genes can lead to tinted eggs in white lines. Genetic analysis has implicated dominance in the shell color formation procession where the dam’s variance component has a stronger effect than that of the sire [5,10,11]. Redman and Shoffner [12] detected dominance based on analyses of covariance related to dams and sires. Other factors might also affect the variations in eggshell color, such as nutrition, disease, and age, but their contribution to shell color is limited [13,14].

In chickens, eggshells are colorful and the lightness of the shell varies substantially within the same breed in some cases. Previous studies indicate that the eggshell color is influenced by dominance in poultry [15], but specific estimates have not been reported for chickens. In this study, we estimated the additive and dominant genetic effects on variations in eggshell color in an F2 population using an animal model analysis.

## MATERIALS AND METHODS

### Birds

The population selected for this study comprised a pure White Leghorn line, which was reciprocally crossed with Dongxiang blue-shelled chickens in an F2 design. These two parents are known to differ in terms of their eggshell traits. In the F1 generation, 49 cocks were mated with 639 hens. The F2 generation group comprised 25 half-sibs and 24 randomly mated families. The original data set comprised 7,878 records for eggshell color in 2,626 individual fowl. Only the most extreme values were removed from the analysis (i.e., values that varied by +3 and −3 standard deviations from the mean), whereas the moderate extreme values were retained as recommended by Hunter and Schmidt [16]. During the first week after hatching, all chicks were provided with artificial illumination throughout the night. The photoperiod was then decreased by 1 h/wk until 9 h of light was provided. Pullets were exposed to natural light until they were transferred into single-hen cages at 16 weeks of age. The light treatment was gradually increased by 1 h per week until 16 h of light was provided. The laying mash contained 16.5% crude protein and it provided 2,750 kcal of metabolizable energy/kg.

Eggs were collected on five consecutive days in week 32. Shell colors were measured daily and the average for three or two eggs (if a hen laid only two eggs) was used as the value for each hen. The International Commission on Illumination L*a*b* color scale, where L* = lightness (100 = white and 0 = black), a* = redness (green is toward the negative end of the scale and red towards the positive end), and b* = yellowness (blue is toward the negative end and yellow toward the positive end of the scale), was used to determine the egg colors with a Portable Spectrophotometer CM-2300D (Minolta Cameras, Osaka, Japan).

### Statistical analysis

The genetic and environmental components incorporated in the model were designed to reflect the data structure and they were inferred from previously reported genetic evaluations of eggshell color [13,17,18]. Data were analyzed with a univariate animal model with an inverting dominance relationship matrix. As suggested by Hoeschele and van Raden, the mixed model included the individual inbreeding coefficients as fixed effects [19]. The inbreeding coefficient for each animal in the pedigree was calculated using ENDOG software [20]. The matrix notation for the model is as follows:

$$y=Xb+Z_{a}a+Z_{d}d+e,$$

where *y* represents an n×1 vector of phenotypes for all the individuals, *X* represents an n×c matrix of c covariates, *b* is a c×1 vector of the effects of the covariates, *a* comprises the additive genetic effects, *d* comprises the dominant genetic effects, *Za* and *Zd* are incidence matrices that relate the animals in the pedigree to records for the additive and dominance effects, respectively, and *e* is the residual (Co) variance matrix. The means and variances in the dominance model are as follows:

$$\begin{array}{l} y|b,a,d,e\sim N(Xb+Z_{a}a+Z_{d}d,R),\\ \text{and }\left[\begin{array}{c} a\\ d\\ e \end{array}\right]\sim N(0,V). \end{array}$$

$$V=\left[\begin{array}{ccc} A\sigma_{a}^{2} & 0 & 0\\ 0 & D\sigma_{d}^{2} & 0\\ 0 & 0 & I\sigma_{e}^{2} \end{array}\right],$$

where A and D are the additive and dominant animal relationship matrices, respectively.

A normal distribution was assumed for all the random effects in all of the models, and the commonly used prior specification for variance components (inverse-Wishart, V = 1, nu = 0.002) was used for the preliminary analysis. However, the autocorrelations were too strong for dominance and residual variance. The residual was fixed as 1 in the analysis of shell lightness. For each model, one chain was run with 500,000 iterations where the first 20,000 were discarded as a burn-in. The chain was thinned by 100 to yield 4,800 samples from the posterior distribution. Genetic and phenotypic correlations were conducted by bivariate analysis. Analyses were run in R version 2.10.1 (R Development Core Team, Vienna, Italy) using the MCMCglmm, version 2.25 and nadiv version 2.14.3.1 packages [21,22]. The deviance information criterion (DIC) was used to detect significant effects of the additive and dominance genetic components of the models. The statistical significance of the genetic estimates (i.e., how much they differed from zero) was assessed by using 95% confidence intervals for the heritability estimates, and the dominance effects were calculated from the posterior distributions [5].

## RESULTS

**Figure 1:**
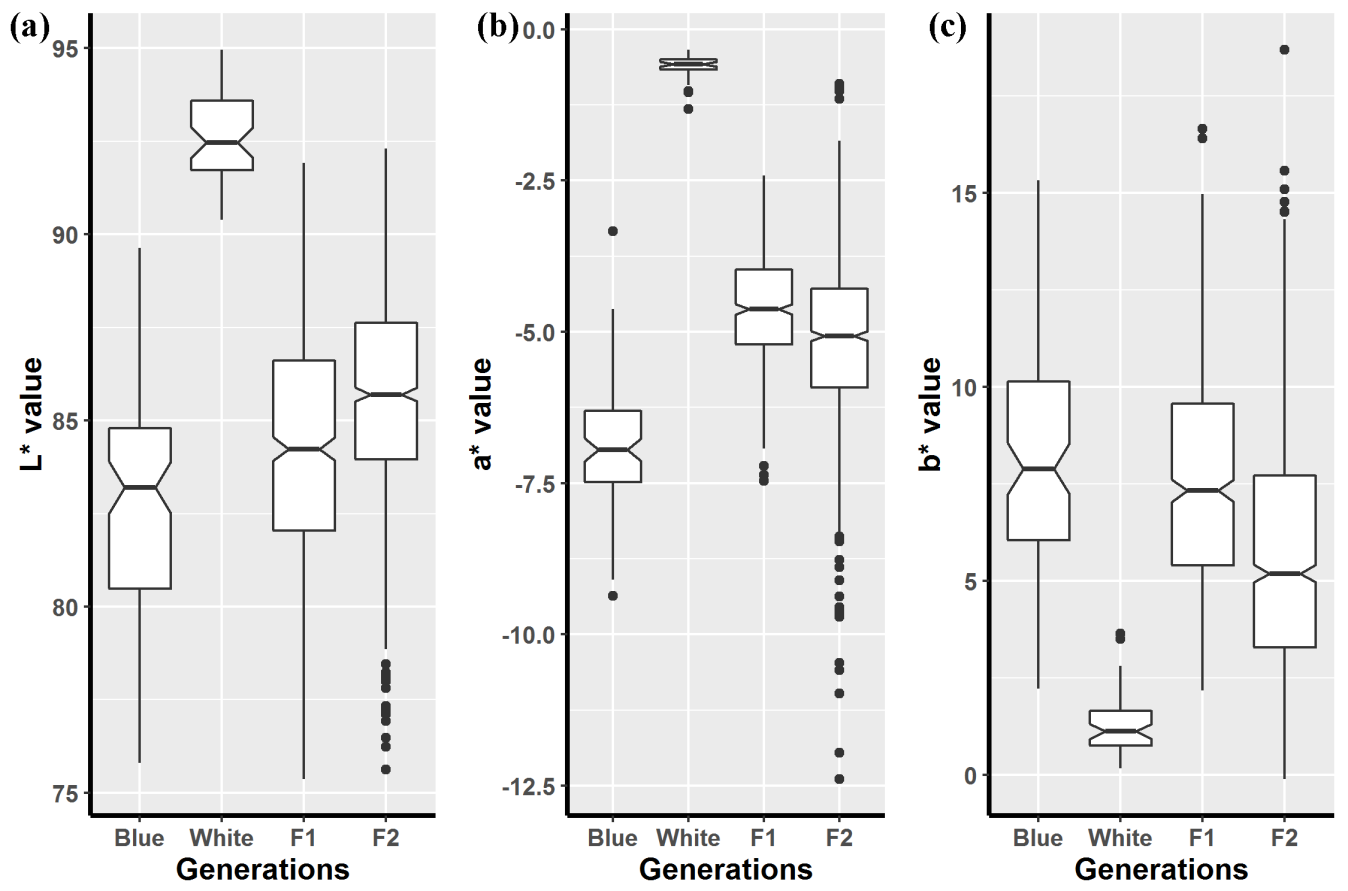
Boxplots of shell color measurements after 32 weeks in a crossbred population. Horizontal lines within each box plot denote the middle values and the circles outside the top/bottom are the mild extreme values. The measurements express as: (a) the lightness, (b) the redness, (c) the yellowness.

After data cleaning, the data set comprised 7,821 records for 72 sires and 787 dams. Significant differences were tested between the parents with respect to the mean L*, a*, and b* values (Figure 1). In the F1 generation, the lightness (L* value) and yellowness (b* value) were close to those for blue-shelled chickens, and the redness (a* value) was in the middle near the mean for the parents. The coefficients of variation for the L*, a*, and b* values increased in the F2 generation compared with those in the parents and F1 generation. The normality of the shell color distribution was examined using the Kolmogorov–Smirnov test (p<0.01). For the birds in this study, the individual inbreeding coefficients ranged from zero to 25% and the frequency distribution had a long tail. The mean and median inbreeding coefficients in the F2 generation were 7.88% and 3.13%, respectively. The effective population size was 9.42.

The results in Table 1 show that the goodness of fit differed for the models in terms of the dominant effects. Based on the DIC values, a model with additive and dominant effects was sufficient to evaluate the shell color with respect to the L* value. The DIC values for models based on the a* and b* measurements differed little with or without dominance effects. According to Ockham’s razor strategy, we tested the additive model as the candidate. Preliminary tests with a covariate comprising the inbreeding coefficients detected significance differences for the fixed effect (p<0.01).

**Figure 2:**
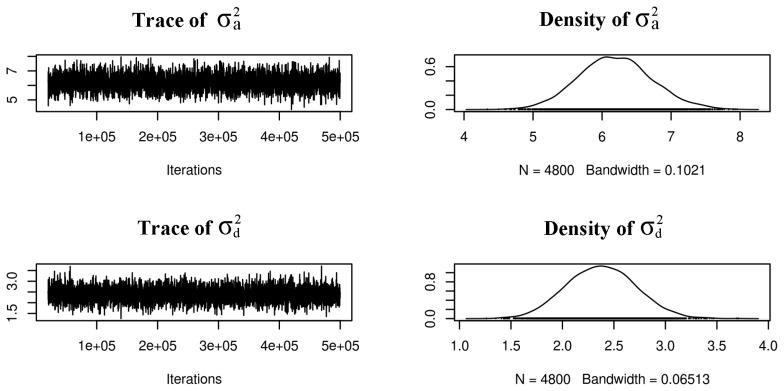
Monte Carlo Markov chain realizations of the variance parameters in a shell lightness data. On the right panel the posterior probability density plot of the additive and dominant variance respectively.

**Figure 3:**
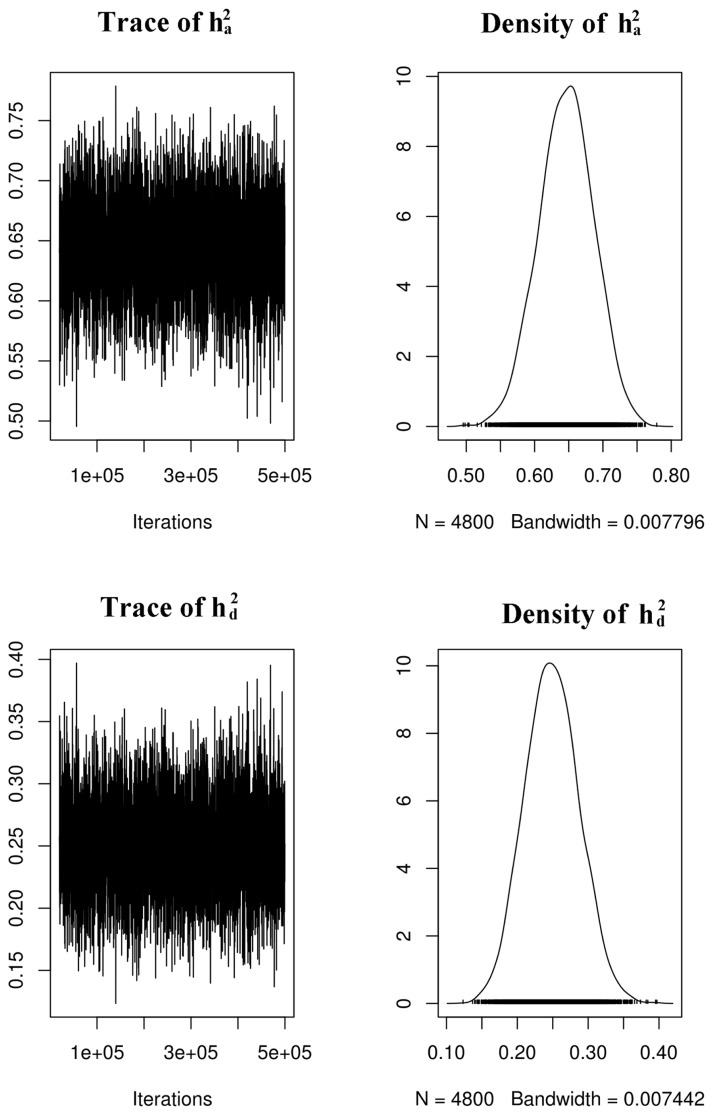
Trace and posterior density plots of additive ($h_{a}^{2}$) and dominance ($h_{d}^{2}$) heritability on the shell lightness.

The estimated heritabilities of eggshell color are shown in Table 2, Figure 2 and 3. The model based on the L* value, which treated it as a random effect with additive and dominant genetic influences, had higher narrow heritability but the differences among the three traits were small compared with the overlapping confidence interval. The additive variances estimated for the three traits were close to each other.

The autocorrelation was slightly high for the residual of the a* value (lag 100 = 0.16). However, the result was accepted considering the unimodal distribution of the posterior density (Figure 2, Supplementary S1, S2). The other autocorrelations were less than 0.10, which implied that the mixing of the Monte Carlo Markov chain (MCMC) was good.

Table 3 shows the phenotypic and genetic correlations between eggshell colors after 32 weeks. The estimated phenotypic correlations differed and the genetic correlations indicated moderate to strong relationships. The genetic and phenotypic correlations were highly negative, where they indicated an antagonistic relationship between the b* value and L* value. There were negative genetic relationships between the b* value and the other measurements.

## DISCUSSION

In the Chinese market, customers increasingly favor blue eggs. Thus, a breeding strategy needs to be developed to improve the uniformity and persistence of the shell color in blue-shelled layers. In this study, we estimated the genetic parameters for shell color using dominance and additive models. The results showed that a dominance effect played an important role in the deposition of shell pigments.

Moderate to high estimates of heritability for shell color would indicate a range for efficient selection. We also compared our estimates with those obtained in previous studies. The heritability of the L* value appeared to be large compared with the heritability estimates reported by Cavero et al [4] (Rhode Island Red = 0.46) and Goger et al [23] (Rhode Island Red = 0.55). However, they analyzed data collected from a brown-egg population whereas we used blue-white crossed layers. Our estimate is close to the values obtained for Barred Rock (0.62) [23] and Hy-line chickens (0.67) [24]. Previous estimates of the heritability of shell lightness ranged from 0.27 to 0.58, but they were measured using a reflectometer [11,12,17]. We found that the estimated heritability of the a* value was the lowest among the three measurements, where the value is similar to the estimate obtained by Cavero et al [4]. (Rhode Island Red = 0.43). Goger et al [23] also used Rhode Island Red lines to estimate genetic parameters based on the a* value and the estimated heritability was 0.51. In the same study, the heritability was found to approach 0.32 for Barred Rock. Our estimate of heritability for the b* value is similar to those reported by Goger et al [23], but larger than those reported by Cavero et al [4]. It appears that the b* value is likely to be influenced by the genetic background.

In addition to the redness and yellowness, we explored the variance in dominance based on lightness measurements obtained in the animal model. The results agreed with the biochemical and molecular biological analyses. It is well known that protoporphyrins and biliverdins are responsible for brown and blue shells, respectively. In particular, protoporphyrins are highly concentrated in the external shell layers or in the cuticle, whereas biliverdin is distributed throughout the shell structure [25,26]. Moreover, Wang et al [27] reported that the amount of eggshell pigments in the eggs of blue-shelled chickens does not differ significantly from that in those of brown-shelled chickens such as Dongxiang chickens. Thus, the shell lightness depends on biliverdins and their derivatives, where the presence of more biliverdins will lead to a larger L* value. The genetic architecture of biliverdin formation in shells is associated with a retrovirus insertion, which led to the expression of the solute carrier organic anion transporter family member 1B3 gene in the shell gland [28]. The blue shell gene is dominant with respect to the white shell gene in chickens. Therefore, it was reasonable to identify variations in dominance in the white-blue shelled cross population.

Genetic correlations can be used to manage selection de cisions regarding specific traits. In this study, we examined the genetic and phenotypic correlations among the three shell color measurements. We compared our results with those obtained by Goger et al [23] but there were no similarities. They found a negative correlation between the L* and a* values, whereas we detected a positive correlation. The b* values and the other measurements were negatively correlated in the present study. A negative correlation was also identified between the L* and b* values in the previous study but it was weaker, whereas the a* and b* values were positively correlated. These differences may be explained by the specific genetic backgrounds considered, where the previous study used brown-shelled chickens and only one shell pigment was investigated. The present study considered white, blue, and tinted egg, as well as protoporphyrins and biliverdins.

## CONCLUSION

In the present study, our genetic evaluation of eggshell color indicated that the shell lightness was under the control of dominant genetic factors. The estimated heritability was reduced compared with the model only with additive effects but the reliability of the estimated breeding values would be increased by the introduction of dominance effects. However, more research is required to estimate the advantages of incorporating a genetic dominance effect in the statistical model for a pure line. In this study, the variance in genetic dominance was analyzed in blue-shelled chickens, but this does not mean that a similar dominant effect will be identified in brown-shelled chickens. In practice, genetic effects may vary between populations, and thus it is important to investigate chickens with brown and white eggshells. This study showed that genetic dominance effects are important for variations in eggshell color, and these findings may have potential applications in mating systems.

## Figures and Tables

**Table 1:** Deviance information criterion values for eggshell color submodels

Item	Additive model	Dominance model
L* value	11,440.07	9,590.06
a* value	13,421.21	13,409.5
b* value	13,748.96	13,748.58

**Table 2:** Variances and heritabilities of eggshell color

Traits	$\sigma_{a}^{2}$	$\sigma_{d}^{2}$	$\sigma_{e}^{2}$	$h_{a}^{2}$	$h_{d}^{2}$
L* value	6.19 (5.14–7.22)	2.38 (1.75–3.05)	1	0.65 (0.57–0.72)	0.23 (0.18–0.33)
a* value	5.55 (3.44–7.64)	-	7.31 (6.04–8.57)	0.42 (0.29–0.56)	-
b* value	10.82 (7.58–14.14)	-	7.37 (5.50–9.06)	0.60 (0.47–0.72)	-

**Table 3:** Genetic and phenotypic correlations with shell color

Items	L* value	a* value	b* value
L * value	-	0.110±0.024	−0.672±0.013
a* value	0.612±0.079	-	0.365±0.020
b* value	−0.844±0.033	−0.387±0.129	-
