# Supplementary material for: Genetic evaluation of eggshell color based on additive and dominance models in laying hens
Source: Asian-Australas J Anim Sci. 2019 Aug 26;33(8):1217–23. doi: 10.5713/ajas.19.0345 (PMC7322644; doi:10.5713/ajas.19.0345)
Supplement: Supplementary file 1 [file ajas-19-0345-suppl1.pdf]

Supplemental materials:

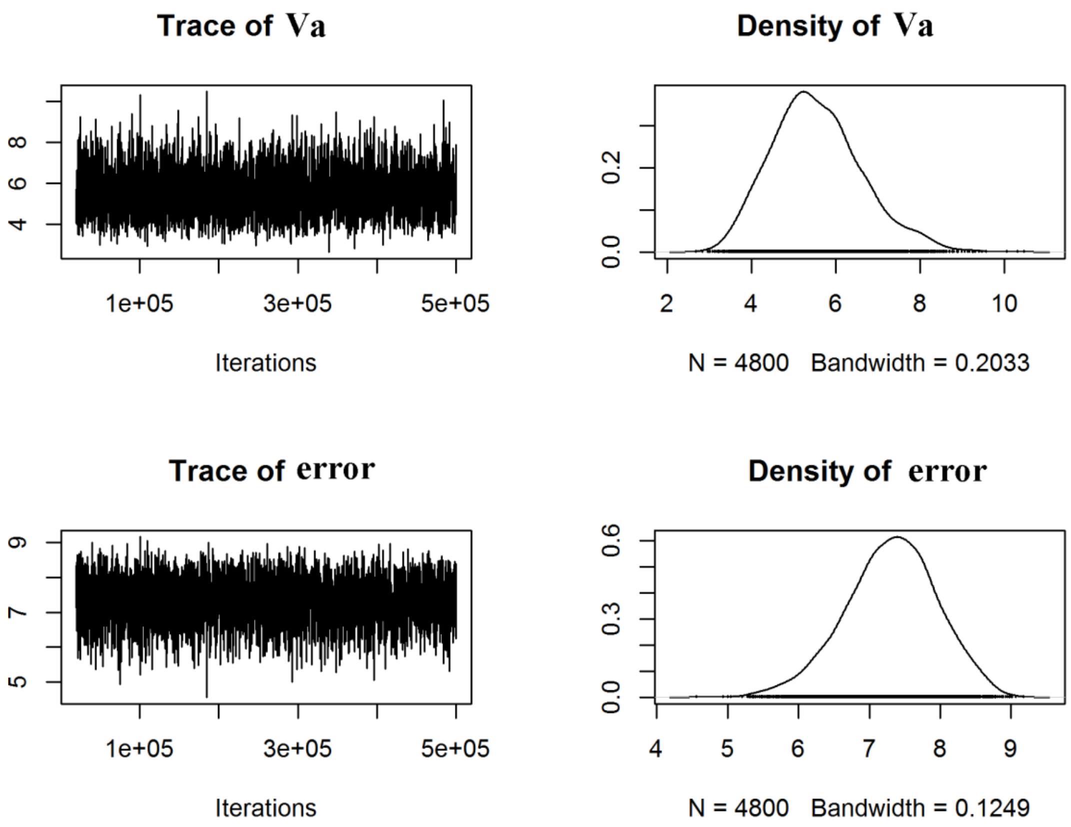

Supplementary Figure S1. MCMC realizations of the variance parameters in a shell redness data. The panels illustrates the trace and probability density plot of the additive (up) and residual(down) variance respectively.

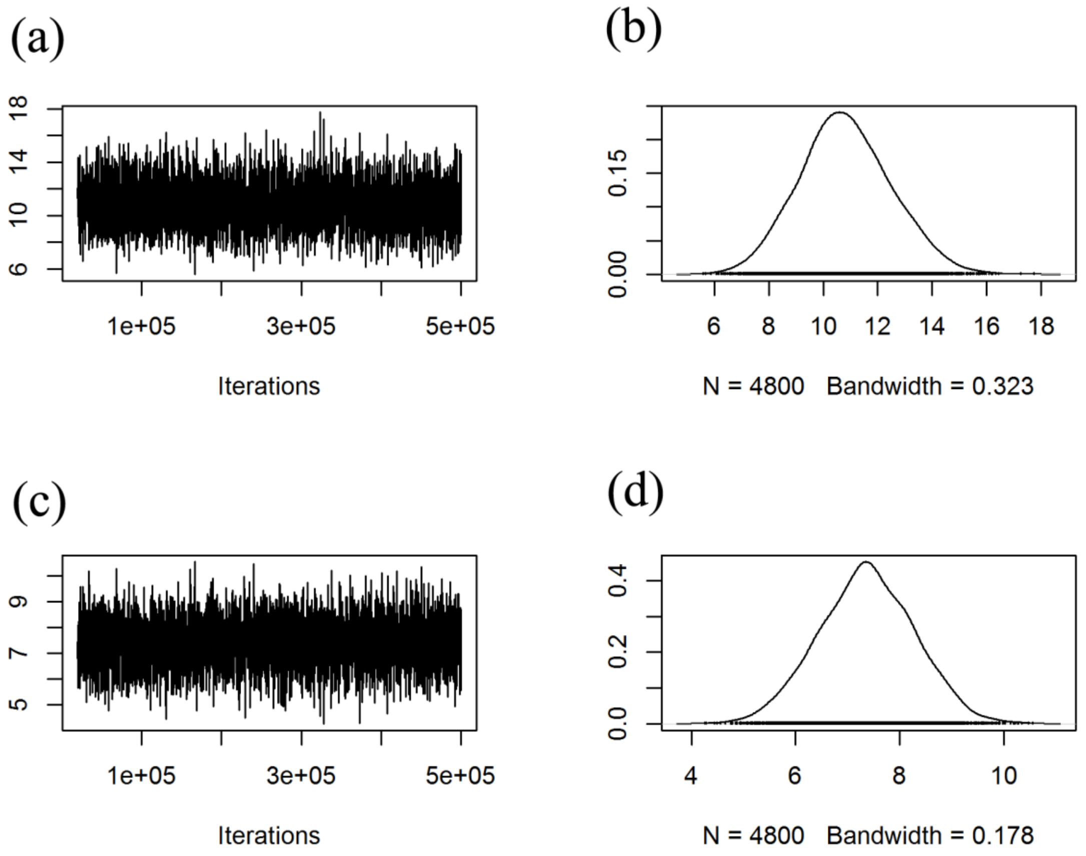

Supplementary Figure S2. MCMC realizations of the variance parameters in a shell yellowness data. The panels illustrates the trace and probability density plot of the additive (up) and residual(down) variance respectively.
